# Hereditary Coproporphyria Mimicking Guillain-Barré Syndrome After COVID-19 Infection

**DOI:** 10.7759/cureus.21586

**Published:** 2022-01-25

**Authors:** Margaret Upchurch, Jonathan P Donnelly, Emily Deremiah, Colleen Barthol, Shaheryar Hafeez, Karl E Anderson, Ali Seifi

**Affiliations:** 1 Department of Neurology, University of Colorado Anschutz Medical Campus, Denver, USA; 2 Department of Neurology, University of Texas Health Science Center at San Antonio, San Antonio, USA; 3 Department of Critical Care Pharmacy, University of Texas Health Science Center at San Antonio, San Antonio, USA; 4 Department of Neurosurgery, University of Texas Health Science Center at San Antonio, San Antonio, USA; 5 Department of Gastroenterology, University of Texas Medical Branch at Galveston, Galveston, USA

**Keywords:** acute flaccid paralysis, givosiran, guillain-barre syndrome (gbs), porphyria, covid-19

## Abstract

Hereditary coproporphyria (HCP) is a rare disorder caused by a deficiency of an enzyme, coproporphyrinogen oxidase, in the heme synthetic pathway. This disease has a highly variable clinical presentation with acute attacks of neurologic symptoms that can last from days to months. Rarely, it and other acute porphyrias may cause ascending paralysis, which is difficult to distinguish from Guillain-Barré syndrome (GBS). Acute attacks can be triggered by factors that increase the synthesis of heme, such as hormonal changes, certain medications, dietary changes, and infections.

We report a 26-year-old female with HCP who presented with acute ascending flaccid paralysis and respiratory failure after coronavirus disease 2019 (COVID-19) infection and was initially misdiagnosed and treated for GBS. She was transferred to our neurosciences intensive care unit, where the diagnosis of acute porphyria was established. Initial improvement occurred during treatment for several weeks with hemin (Panhematin®) and continued with givosiran (Givlaari®), which was recently introduced for the prevention of acute attacks.

We suggest that acute porphyria should be part of the differential diagnosis when GBS is suspected. To our knowledge, this is the first report of an attack of acute hepatic porphyria (AHP) that developed after a COVID-19 infection and the first with advanced paresis to be treated with givosiran. Her response suggests that givosiran may contribute to recovery from advanced neurological manifestations of acute porphyrias.

## Introduction

Porphyrias are a family of rare disorders that result from the altered activity of enzymes involved in heme synthesis. The four acute hepatic porphyrias (AHP) are characterized by neurological manifestations and elevation of δ-aminolevulinic acid (ALA), porphobilinogen (PBG), and porphyrins due to their overproduction by the liver. This occurs when certain triggering factors induce δ-aminolevulinic acid synthase 1 (ALAS1), the initial and rate-limiting enzyme of the heme synthetic pathway in the liver, causing an inherited partial deficiency of a subsequent pathway enzyme to become rate-limiting. Hereditary coproporphyria (HCP) is caused by a partial deficiency of coproporphyrinogen oxidase (CPOX), the sixth enzyme in the heme synthetic pathway. This is an autosomal dominant genetic disorder with variable penetrance that typically presents after puberty with attacks of neurovisceral symptoms, and much less commonly with cutaneous photosensitivity. Rarely, a progressive motor neuropathy in this and other AHPs can resemble Guillain-Barré syndrome (GBS). The rarity of AHP and the nonspecific nature of its symptoms often result in delays in diagnosis and initiation of specific treatment.

Neurovisceral attacks are generally treated with hemin. Givosiran (Givlaari®, Alnylam, Cambridge, MA) is a newer medication approved for the treatment of AHP and is given via monthly injection to prevent porphyria attacks. It functions by reducing the levels of ALAS1 in hepatocytes.

We describe a case of a young woman with a severe attack of AHP after coronavirus disease 2019 (COVID-19) infection, with progression to widespread flaccid paralysis and respiratory depression before she was found to have HCP. Treatment with intravenous hemin resulted in improvement. This is the recommended first-line treatment for acute attacks and acts by repleting heme deficiency, thereby repressing synthesis of hepatic ALAS1 and reducing a potentially neurotoxic buildup of ALA and PBG. Hemin is relatively short-acting and may require daily administration. Recovery continued with a monthly injection of givosiran, a long-acting small interfering RNA (siRNA) directed against ALAS1 mRNA in the liver, also lowering ALA and PBG.

## Case presentation

A 26-year-old female developed COVID-19 infection with mild respiratory and gastrointestinal symptoms. Past history included anxiety and allographic renal transplant at age 21 for presumed idiopathic glomerulonephritis. During recovery at home four weeks later, she developed progressively worsening abdominal pain accompanied by burning dysesthesias in the lower and then the upper extremities. Symmetric, distal-predominant, and ascending weakness developed four weeks later, associated with severe holocephalic headaches and complex visual hallucinosis. Evaluation at an outside hospital included magnetic resonance imaging (MRI) of the brain and spine with and without contrast, which revealed no acute abnormalities. Cerebrospinal fluid (CSF) glucose was 56 mg/dL, protein level was 45.5 mg/dL, white blood cell count was 5/mcL, and red blood cell count was 258/mcL. A course of intravenous immunoglobulin (IVIG) was given for presumed GBS, but symptoms progressed to neuromuscular respiratory failure with her negative inspiratory force (NIF) dropping from −40 to 0 cm H2O. When transferred to our tertiary care neuroscience critical care unit (NSICU), her treatment included anesthetic and paralytic agents, tacrolimus 1 mg twice daily, prednisone 5 mg once daily, and atorvastatin 40 mg once daily.

The initial exam showed a Glasgow Coma Scale (GCS) score of eye (E) 4, T (intubated), and motor (M) 6, heart rate of 109 beats per minute, and blood pressure of 126/95 mmHg. The abdomen was distended and tender in all four quadrants, but without rebound tenderness or guarding and with normoactive bowel sounds. There were no skin lesions. The patient was alert and oriented to person, place, time, and situation. Cranial nerves were normal except for bilateral ophthalmoplegia. The tone was diminished throughout, with reduced muscle bulk in both lower extremities. Strength was symmetric 0/5 in the lower and proximal upper limbs, with some preservation of distal finger flexion (1/5). Sensation to light touch was decreased focally in a right C6/7 and bilateral proximal lower extremity distribution. Deep tendon reflexes (DTRs) were absent throughout. Coordination and gait could not be assessed. Summarized initial laboratory studies, including autoimmune and infectious testing, are outlined in Table 1.

**Table 1:** Summarized hematologic and biochemical lab results. Abnormal values are in bold.

Laboratory test	Value	Normal ranges
Hemoglobin (Hb)	8.3 g/dL	11.5-14.9 g/dL
Hematocrit (HCt)	24.9%	36.0-45.5 %
White blood cell count (WBC)	4.96 K/mcL	3.40-10.40 K/mcL
Platelet count (Plt)	78 K/mcL	140-377 K/mcL
Erythrocyte sedimentation rate (ESR)	39 mm/hr	2-37 mm/hr
Creatinine	2.27 mg/dL	0.50-1.10 mg/dL
Blood urea nitrogen (BUN)	67 mg/dL	7-25 mg/dL
Alanine aminotransferase (ALT)	83 U/L	<36 U/L
Aspartate aminotransferase (AST)	95 U/L	<31 U/L
Albumin	1.7 g/dL	3.2-5.0 g/dL
Vitamin B12	1,095 pg/mL	247-911 pg/mL
Thyroid-stimulating hormone (TSH)	3.863 mcIU/mL	0.350-5.500 mcIU/mL
Antinuclear antibody (ANA)	Not detected	N/A
Extractable nuclear antigens (ENA)	Not detected	N/A
Ganglioside antibodies (GM1, GM2, GD1a, GD1b, GQ1b)	Negative	N/A
Lupus anticoagulant	Negative	N/A
Human immunodeficiency virus (HIV) screen	Negative	N/A
Epstein-Barr virus, plasma	Not detected	N/A
Cytomegalovirus polymerase chain reaction (PCR)	<137 IU/mL	<137 IU/mL
Borrelia burgdorferi IgG/IgM	Negative	N/A
Acute hepatitis (A, B, C) panel	Negative	N/A

COVID-19 testing was negative, indicating recovery from the infection. Additional infectious workup, including stool ova and parasite testing, was negative, except our local community-acquired diarrhea panel, which was positive for enteropathogenic *Escherichia coli*. Repeat MRI of the brain and whole spine with and without contrast was negative.

Electrodiagnostic testing was consistent with profound axonal sensorimotor peripheral polyneuropathy affecting all extremities. Findings included the absence of sensory nerve action potentials, prolonged F wave latencies, and very low amplitude compound motor action potentials. Needle electromyography (EMG) suggested acute or subacute denervation in all muscles tested. Specific values are outlined in Tables 2, 3.

**Table 2:** Patient's nerve conduction study results. NR indicates no response. * indicates abnormal value. F waves were also absent in the right ulnar and bilateral tibial nerves. Although F wave absence is classically associated with Guillain-Barré syndrome, this was likely related to the severely reduced compound muscle action potential (CMAP) amplitudes. The lack of conduction block or temporal dispersion is not in keeping with demyelinating disease.

Sensory nerve conduction studies					
Nerve	Site	Distance	Peak latency	Amplitude	Nerve conduction velocity (NCV)
Right ulnar	Wrist	14 cm	NR	NR	N/A
Right sural	Ankle	14 cm	NR	NR	N/A
Left sural	Ankle	14 cm	NR	NR	N/A
Motor nerve conduction studies					
Right ulnar	Wrist	8 cm	3.5 ms	0.28 mV*	
	Below elbow	NR	NR	NR	N/A
Right tibial	Ankle	8 cm	3.5 ms	0.22 mV*	
	Popliteal fossa	34 cm	10.6 ms	0.27 mV	48 m/s
Left tibial	Ankle	8 cm	4.7 ms	0.52 mV*	
	Popliteal fossa	35 cm	11.5 ms	0.54 mV	51 m/s

**Table 3:** Patient's needle electromyography results. The reduced recruitment suggests axonal denervation, again not in keeping with demyelinating disease, although could potentially be seen in acute motor axonal neuropathy (AMAN). PSW - positive sharp waves; fibs - fibrillation potentials; MUAPs - motor unit action potentials.

Electromyography				
Muscle	Insertional activity	Rest activity	Motor unit action potential (MUAP)	Recruitment
Left deltoid	Increased	PSWs, fibs	Not observed	Unable to recruit MUAPs for evaluation
Left triceps	Increased	PSWs, fibs	Not observed	Unable to recruit MUAPs
Left 1^st^ dorsal interosseous	Increased	PSWs, fibs	Not observed	Unable to recruit MUAPs
Left tibialis anterior	Increased	PSWs, fibs	Not observed	Unable to recruit MUAPs
Left medial gastrocnemius	Normal	Silent	Not observed	Unable to recruit MUAPs
Left vastus medialis	Increased	PSWs, fibs	Not observed	Unable to recruit MUAPs

These results were not typical of GBS but suggested acute motor and sensory axonal neuropathy (AMSAN), and plasma exchange (PLEX) was initiated on hospital day four. Due to persistent respiratory failure, a tracheostomy was inserted on hospital day five, and a percutaneous endoscopic gastrostomy (PEG) tube on day 16. Her course was complicated by dysautonomia, cardiac arrest and resuscitation, recurrent multi-drug resistant pseudomonas infection, posterior reversible encephalopathy syndrome (PRES), and gastric perforation with peritonitis and septic shock.

Numerous aspects were incongruent with GBS including failure to respond to treatment, normal CSF protein, and atypical EMG findings. Urine porphobilinogen (PBG) was reported at 42.8 umol/L (reference: 0-8.8 umol/L) on a somewhat dilute urine sample (creatinine: 0.61 g/L), establishing a diagnosis of either acute intermittent porphyria (AIP), variegate porphyria (VP), or HCP. Urine porphyrins were also markedly elevated, with a predominance of uroporphyrin and coproporphyrin. Genetic testing to determine the type of AHP revealed heterozygosity for a “variant of uncertain significance” in CPOX, specifically c.1070G>A (p.Cys357Tyr), and no mutation of genes associated with other AHPs, namely, hydroxymethylbilane synthase (HMBS), protoporphyrinogen oxidase (PPOX), and δ-aminolevulinic acid dehydratase (ALAD). There was no family history of porphyria or similar symptomatology. In addition, to our knowledge, the patient was not recently started on medications (including COVID-19 treatments) that could be implicated in causing the AHP attack and did not have any clear triggers (dietary changes, alcohol/drug intoxication, and menstrual cycle). Therefore, the recent COVID-19 infection was presumed to be the precipitating factor for this acute presentation. On hospital day 18, treatment with hemin (Panhematin®, Recordati, Milan, Italy) at 3 mg/kg daily for four days was initiated, which ultimately associated with marked improvement in the patient’s sensory impairment, ophthalmoplegia, abdominal pain, and anxiety, as well as improvement in upper extremity strength to 2/5, hip flexion to 2/5, and knee flexion to 1/5. Urine PBG decreased to <3 umol/L after completion of hemin. The patient received seven more doses over four weeks, a total of eight infusions.

Following stabilization of the patient’s condition, on hospital day 105, treatment with monthly givosiran (Givlaari®, Alnylam) 2.5 mg/kg subcutaneously monthly was started to prevent further exacerbations. The patient's condition at this point in her hospital stay is shown in Video 1.

**Video 1:** The patient's condition prior to starting givosiran (hospital week 16). At this point, she is still requiring ventilatory support via tracheostomy and has 2/5 strength in the distal upper and lower extremities. The patient provided signed informed consent for the use of this video.

After starting this therapy, our patient experienced significant progressive gains in her motor function during physical therapy. Tracheostomy and PEG tube were removed, and physical therapy cleared her discharge for outpatient care. After 183 days in the hospital, the patient was able to stand with assistance and mobilize with supervision and was discharged home.

The patient continued to improve at home with nearly full recovery of motor function and no respiratory problems. She became able to walk unassisted with only some weakness in ankle plantar and dorsiflexion and mild lower limb dysesthesias. She is currently doing therapy at home on her own while waiting for insurance approval of home physical and occupational therapy. Her most recent physical condition is shown in Video 2.

**Video 2:** The patient's most recent physical condition approximately one year after discharge from the hospital. She is able to walk unassisted with bilateral ankle-foot orthoses, with some residual bilateral foot drop and lower extremity dysesthesias. The patient provided signed informed consent for the use of this video.

Monthly givosiran treatment continues, with careful kidney and liver test monitoring. She is on dialysis three times a week and will likely receive another renal transplant in the future.

## Discussion

Acute flaccid paralysis has a broad differential, ranging from infectious (with paresis developing during or after infection), autoimmune, vascular, toxic/metabolic, and many other disorders including AHP [1]. Now that polio is prevented by widespread vaccination, GBS and its variants have become the most common causes in adults. GBS is thought to be due to molecular mimicry, with an inciting illness or trauma leading to the production of autoantibodies that attack targets such as gangliosides in the peripheral nervous system [2]. The clinical presentation of GBS is variable and can be preceded by a number of viral and bacterial infections [2]. It typically begins with acroparesthesia, followed by ascending weakness and areflexia and often progresses over hours to days. Diagnosis of GBS is supported by CSF findings of albuminocytologic dissociation (elevation of protein without significantly raised white cell count), and electrodiagnostic findings consistent with demyelination or, in some variants, axonal loss. Treatment is usually with IVIG, and rehabilitation, with recovery taking place usually over several months [3]. Presenting features of GBS are not specific, and it is increasingly recognized that AHP should be considered in the differential diagnosis [4-6]. AHP may not be suspected because it is rare and the symptoms and signs are nonspecific, even when severe, so testing is appropriate even when the level of suspicion is low. It is unfortunate that rapid testing for PBG, which can readily rule the diagnosis in or out, is no longer available at most medical centers [7]. In the case described here, testing for elevated urine PBG enabled a diagnosis for which treatment with hemin was available without further delay, and led to improvement in neurological function.

Porphyrias are rare metabolic disorders each resulting from the altered activity of one of the eight enzymes involved in heme synthesis [7]. Heme synthesis occurs in all tissues to form essential hemoproteins. The bone marrow and liver synthesize heme in the largest amounts to supply large amounts of heme for hemoglobin and cytochrome P450 enzymes, respectively. Porphyrias are classified as hepatic or erythropoietic depending on whether an accumulation of intermediates of the pathway occurs initially in the liver or bone marrow. Heme is synthesized in smaller amounts in all other tissues, including the nervous system. The four AHPs cause neurological manifestations, usually occurring as acute attacks during adult life, with an elevation of the porphyrin precursors ALA and PBG as well as porphyrins [7]. ALA and PBG have not been conclusively shown to explain neurotoxicity in AHPs. Alternative hypotheses include heme deficiency in the nervous system or blood vessels, the latter potentially causing deficiencies of key hemoproteins such as nitric oxide (NO) synthase, resulting in vasospasm [8,9].

Abdominal pain is the most common presenting symptom of AHP. It is usually poorly localized and often associated with nausea, vomiting, constipation, bloating, and distension. Since the pain is neurological rather than inflammatory, abdominal tenderness, fever, and leukocytosis are typically absent [7,10]. Other autonomic findings such as tachycardia, hypertension, and sweating are common. Central neurologic manifestations can include insomnia, anxiety, agitation, and hallucinations. Hyponatremia may result from hypothalamic involvement and the syndrome of inappropriate secretion of anti-diuretic hormone (SIADH). Peripheral sensory neuropathy causing extremity, chest, and back pain is common. Axonal motor neuropathy is often a later feature of severe attacks, and usually begins proximally in the upper extremities and, as in our patient, may progress to quadriparesis and respiratory failure [7,11,12]. This presentation can mimic GBS or its variants, in which the characteristic CSF and EMG findings may take several days to develop [6]. Our patient lacked some important features of GBS, such as CSF protein elevation or elements of a motor axonal neuropathy on nerve conduction study, such as temporal dispersion and/or conduction block. Findings more consistent with a toxic/metabolic polyneuropathy included absent sensory nerve action potentials (SNAPs) and low amplitude compound muscle action potentials (CMAPs). The severity of the neurovisceral manifestations was unusual for HCP, which is generally a milder disease than AIP. This may have included PRES, which was attributed to tacrolimus use but can also be associated with AHP [11]. However, it is established that all AHPs can cause the same severe and even fatal neurovisceral manifestations [7].

The diagnosis of AHP was established by finding the marked elevation of urine PBG, which can occur in AIP and VP as well as HCP. Many different mutations have been described in each of these conditions [7]. This patient’s CPOX mutation has not been previously reported or studied in expression symptoms, so it is presently classified as a variant of unknown significance (VUS). However, the severe manifestations of AHP in this patient along with the absence of HMBS or PPOX mutation strongly suggest that this indeed is a pathogenic CPOX mutation. This is further supported by its rarity in normal population databases, and in silico missense predictions that it is damaging. Efforts to identify others in the family with the same mutation are planned.

Symptomatic and supportive management of acute attacks of AHP is important and includes opioids for pain, antiemetics, sedatives, and monitoring for autonomic and motor dysfunction and electrolyte abnormalities [7,11]. Specific treatments that downregulate hepatic ALAS1 include glucose loading, hemin, and givosiran. Glucose loading is considered only for mild attacks. Hemin is available in the US as lyophilized hematin (Panhematin®) or in some other countries as heme arginate (Normosang®, Recordati, Milan, Italy), and is recommended for the treatment of acute attacks [11,13]. It is administered intravenously at doses of 3-4 mg/kg once daily for approximately four days, or longer, if necessary, until symptoms resolve. In five open-label studies of 99 patients with acute porphyrias, hemin (3-4 mg/kg/day once daily or in divided doses not to exceed 6 mg/kg/day) was associated with improvement in symptoms and reduction in pain in 85.5% of patients. Additionally, in a retrospective study of 108 patients with acute porphyrias that included nine patients with HCP, the administration of hemin was associated with improvement in abdominal pain and other clinical manifestations in 74% of patients [10]. Hemin is most effective and leads to more rapid recovery if given early, especially before the onset of severe motor neuropathy, which recovers more gradually. Complete motor recovery can occur, or some distal paresis may persist long term. After recovery from advanced paresis, the clinical course is quite variable ranging from no further attacks to frequently recurring attacks [7]. Mechanisms responsible for neurological damage in AHP remain poorly understood, and a potential benefit of hemin is that it not only lowers ALA and PBG, which are potentially neurotoxic, but also may replete heme pools and restore normal hemoprotein functions [14].

Givosiran (Givlaari®) is a siRNA therapeutic administered monthly as a subcutaneous injection at a standard dose of 2.5 mg/kg and was approved in 2019 for the treatment of AHP [15,16]. It is derivatized with N-acetylgalactosamine for hepatocyte targeting and within these cells is incorporated into the RNA-induced silencing complex and uses the naturally occurring RNA interference mechanisms to specifically target ALAS1 mRNA, thereby preventing the synthesis of the corresponding ALAS1 protein. Urine ALA and PBG decrease substantially soon after givosiran dosing, and this effect persists for at least a month in most patients. In a pivotal phase 3 trial of patients with frequently recurring attacks, the drug was generally well-tolerated, the annualized rate of attacks was 74% lower with givosiran treatment than with placebo, and the need for treatment with hemin was significantly reduced. Common adverse effects included elevated liver transaminases and serum creatinine, nausea, fatigue, injection site reactions, and rarely anaphylaxis [15,17,18]. To date, givosiran has been studied only in AHP patients experiencing four or more attacks per year with the aim of reducing attack frequency and has not been studied for treating attacks or studied in patients with advanced motor neuropathy. Givosiran may impair heme availability in hepatocytes and thereby reduce activities of some hepatic cytochrome P450s that metabolize drugs [15], and it accentuates hyperhomocysteinemia perhaps by impairing the heme-containing enzyme cystathionine-β-synthase [19]. Long-term safety data and information on safety in patients with pre-existing renal or hepatic disease and during pregnancy are not yet available.

AHP in the setting of severe acute respiratory syndrome coronavirus 2 (SARS-CoV-2) infection is not fully understood, due largely to the fact that AHP is a rare disease in and of itself, so there has not been an abundance of reported cases. Therefore, a specific treatment method for these patients has not been established. Some studies have demonstrated a rise in porphyrins in acute COVID-19, although not to the extent of a true acute hepatic porphyria attack [20]. The mechanism for this is not fully understood, though it is purported to be either due to an inflammation-driven heme shortage or hepatic mitochondrial dysfunction in the setting of increased oxidative stress [20]. Though further study in this area may help us to understand the pathophysiology behind COVID-19-induced porphyria attacks, the treatment is likely to be the same as in any AHP attack, i.e., heme. Patients who are vaccinated against COVID-19 may avoid acute porphyria attacks induced by the disease, though more data are needed [21].

Our patient began to recover after the diagnosis of AHP was made and hemin along with symptomatic and supportive treatment was instituted, which led to a marked reduction of PBG levels. Treatment with givosiran was initiated because it is more practical than hemin for long-term administration and requires less frequent dosing. Due to the fact that AHP patients may not have further attacks after recovery from an initial severe attack, it is not always necessary to use this medication, and long-term use is not typically required. In our patient’s case, we plan to discontinue givosiran in the near future and consider restarting if she develops recurrent attacks.

## Conclusions

To our knowledge, this is the first reported example of a severe attack of AHP following COVID-19 infection. It might be expected that this infection, like others, has the potential to trigger attacks of porphyria. Our case also demonstrates that AHP should be considered in patients with acute motor neuropathy, especially when the laboratory and electrodiagnostic testing does not fit with GBS. Acute attacks of AHP are readily diagnosed or excluded by measuring urine PBG and porphyrins, and a diagnosis enables treatment that is not effective in other conditions. Hemin is recommended for the treatment of acute attacks. Givosiran is highly effective for the prevention of frequently recurring attacks, and, in this case, may have contributed to the prolonged recovery of advanced motor neuropathy. As the pandemic continues, we may learn more about how COVID-19 interacts with rarer diseases, as more people are affected. The salient points of this paper with regard to COVID-19 and AHP are to consider AHP in patients who present with neurovisceral signs and symptoms during or after COVID-19 infection, and secondly, to treat these patients the same as in any other porphyria flare. Furthermore, we stress the importance of vaccination against SARS-CoV-2 to patients with known AHP.
